# Colo-rectal Lithobezoar: A Rare Cause of Colonic Obstruction

**Published:** 2015-01-01

**Authors:** Ghulam Mustafa, Muhammad Saleem

**Affiliations:** Department of Pediatric Surgery, The Children’s Hospital and the Institute of Child Health, Lahore, Pakistan.

**Dear Sir,**

Bezoar is the foreign body material in the digestive tract. Primary colonic lithobezoar is rarely reported in childhood and is usually associated with the history of iron deficiency anemia, pica or psycho-social disorders. [1] These cases may present with chronic constipation, recurrent abdominal pain, and painful defecation. Evacuation under anesthesia is the usual treatment, but surgery can be considered if this procedure fails or colonic injury occurs.[2-4]

A 4-year old boy presented with acute constipation for three days. He had a history of recurrent abdominal pain and painful defecation for the last few months. There was the history of pica (crushed pieces of bricks) for one year. He belonged to a poor socio-economic class and was the 3rd among the 4 siblings of the family. On general physical examination, he was mildly pale. On abdominal palpation, there was mild tenderness. A mass was palpable at the hypogastric area. On digital rectal examination, the anal canal was found full of pieces of red brick. Finger couldn’t be negotiated around the mass. X-ray abdomen showed calcified areas diffusely scattered in the abdomen (Fig. 1).

**Figure:**
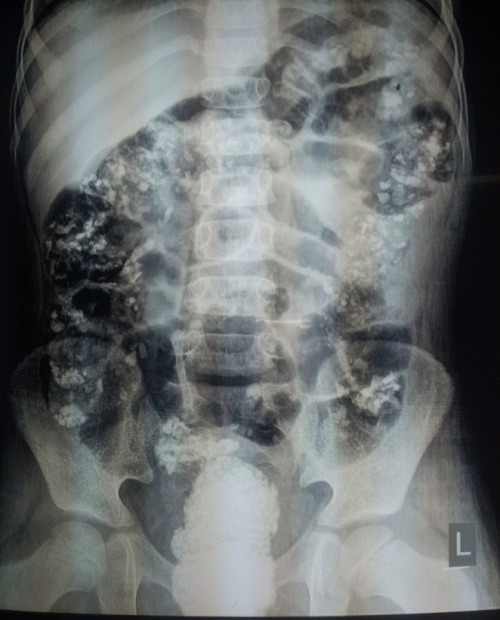
Figure 1: Abdominal radiograph showing multiple opacification of different sizes scattered throughout the large intestine. The rectum is fully calcified showing complete obstruction.

Under general anesthesia lots of small pieces of red brick mixed with feces were manually removed (Fig.2). Colon was washed with saline. Oral laxative was started in postoperative period. He passed few brick pieces for a couple of days. Post-evacuation X-ray, done on 4th postoperative day, showed no opaque areas in the abdomen. Psychiatric consultation showed no gross abnormality. On 5th post evacuation day, the child was discharged on oral iron supplements and with parental counseling as well.

**Figure:**
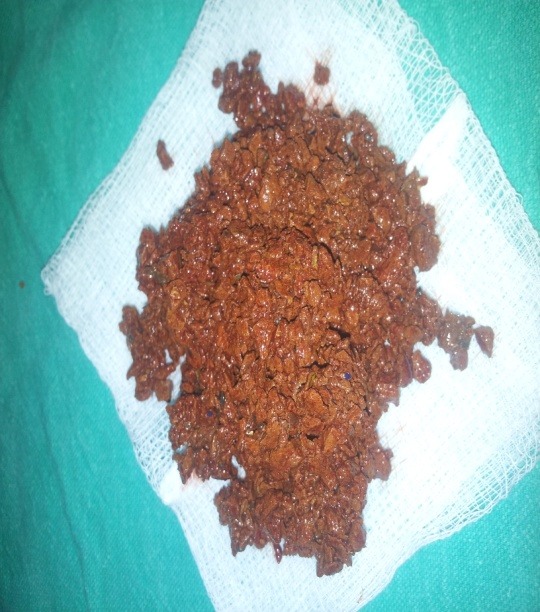
Figure 2: Litho-bezoar evacuated through transanal approach.

Colonic crunch sign is the typical feature of colonic lithobezoar. Colonic crush sign is defined as palpation of spiky mass on digital rectal examination.[5] In our case, abdomen was not distended and lithobezoar had blocked the recto-sigmoid colon. A plain X-ray abdomen in these cases shows scattered radio-opaque shadows throughout the colon which is peculiar of colonic lithobezoar, this peculiar appearance is called corn on cub.

## Footnotes

**Source of Support:** Nil

**Conflict of Interest:** None declared

